# Isolation and characterization of a novel lytic *Parabacteroides distasonis* bacteriophage φPDS1 from the human gut

**DOI:** 10.1080/19490976.2023.2298254

**Published:** 2024-01-04

**Authors:** Adrián Cortés-Martín, Rémi Denise, Emma Guerin, Stephen R. Stockdale, Lorraine A. Draper, R. Paul Ross, Andrey N. Shkoporov, Colin Hill

**Affiliations:** APC Microbiome Ireland & School of Microbiology, University College Cork, Cork, Ireland

**Keywords:** Bacteriophage, *Parabacteroides*, phage-bacteria interaction, phage isolation, fecal fermentation, gut microbiome, phage characterization

## Abstract

The human gut microbiome plays a significant role in health and disease. The viral component (virome) is predominantly composed of bacteriophages (phages) and has received significantly less attention in comparison to the bacteriome. This knowledge gap is largely due to challenges associated with the isolation and characterization of novel gut phages, and bioinformatic hurdles such as the lack of a universal phage marker gene and the absence of sufficient numbers of homologs in viral databases. Here, we describe the isolation from human feces of a novel lytic phage with siphovirus morphology, φPDS1, infecting *Parabacteroides distasonis* APCS2/PD, and classified within a newly proposed *Sagittacolavirus* genus. *In silico* and biological characterization of this phage is presented in this study. Key to the isolation of φPDS1 was the antibiotic-driven selective enrichment of the bacterial host in a fecal fermenter. Despite producing plaques and lacking genes associated with lysogeny, φPDS1 demonstrates the ability to coexist in liquid culture for multiple days without affecting the abundance of its host. Multiple studies have shown that changes in *Parabacteroides distasonis* abundance can be linked to various disease states, rendering this novel phage-host pair and their interactions of particular interest.

## 1. Introduction

Bacteriophages (phages) constitute the majority of the human gut virome and are believed to play an important role in shaping the composition and functionality of the microbiome, either through direct interactions with members of the gut bacterial community, or indirectly by interacting with the host immune system.^1–3^ Several biological factors (i.e., age, diet, geographic location, ethnicity, etc.) and multiple disease states are associated with alterations in the human gut virome composition.^4–7^ For example, in patients with inflammatory bowel disease (IBD) there is a shift in virome composition from predominantly virulent phages toward induction of temperate phages.^8^

Despite this revived interest in phages, the diversity of the gut virome remains understudied. While recent efforts to sequence, assemble, and catalog the genomes of uncultured human gut phages resulted in databases of over 50,000 viral operational taxonomic units (OTUs) representing up to 50% of previously un-annotated gut phage DNA (“the viral dark matter”),^9,10^ most of these genomes remain unclassified and not assigned to any bacterial hosts. The key obstacles include the absence of a universal phylogenetic marker gene in phage genomes, often a lack of sequence homology with known phages included in the present International Committee on Taxonomy of Viruses (ICTV) taxonomy, and the absence of a widely accepted universal framework for classification of novel and uncultured virus taxa.^11,12^ Isolation of novel gut phages in culture can be hampered by: (i) difficulties in culturing host bacteria; (ii) the inadequacy of traditional screening methods involving plaque or spot assays; (iii) an inability to mimic the specific gut conditions required for phage replication; and (iv) the rapid emergence of resistance in bacterial hosts, e.g. through phase variation.^13–15^

The isolation of novel phage-host pairs from the human gut provides new insights into phage-host dynamics and the role of phages in this ecosystem. Order Bacteroidales is one of the most abundant bacterial taxonomic groups in a healthy human gut, with *Bacteroides* and *Parabacteroides* being two of the most abundant genera.^16,17^ Analyses of Clustered Regularly Interspaced Short Palindromic Repeats (CRISPR) spacer sequences, which reveal information about previous encounters between bacteria and genetic elements such as viruses or plasmids, suggest that a significant proportion of persistent virulent phages in the human virome infect hosts of the Bacteroidales order.^18^ The *Bacteroides*-infecting *Crassvirales* (crAss-like phages), the most abundant phage order in the human gut, provide one of the most notable examples of how the isolation and characterization of a phage originally detected *in silico* was key in gaining insights into its biological properties.^19–21^

Here, we conducted an *ex vivo* selective antibiotic enrichment of a fecal bacterial community to promote the growth of Bacteroidales and to permit the parallel expansion of associated phages. We report the isolation of a novel *Parabacteroides distasonis* phage; φPDS1, which is classified in the newly candidate genus *Sagittacolavirus*. This phage is the first lytic *P. distasonis*-infecting siphovirus isolated from human feces to be characterized and to have its genome sequenced. Given that *P. distasonis* has been linked to certain human diseases,^17,22^ characterization of its phages takes on an added significance.

## 2. Results

### 2.1. Bacterial composition in fecal fermentation following antibiotic enrichment

**Figure 1.**
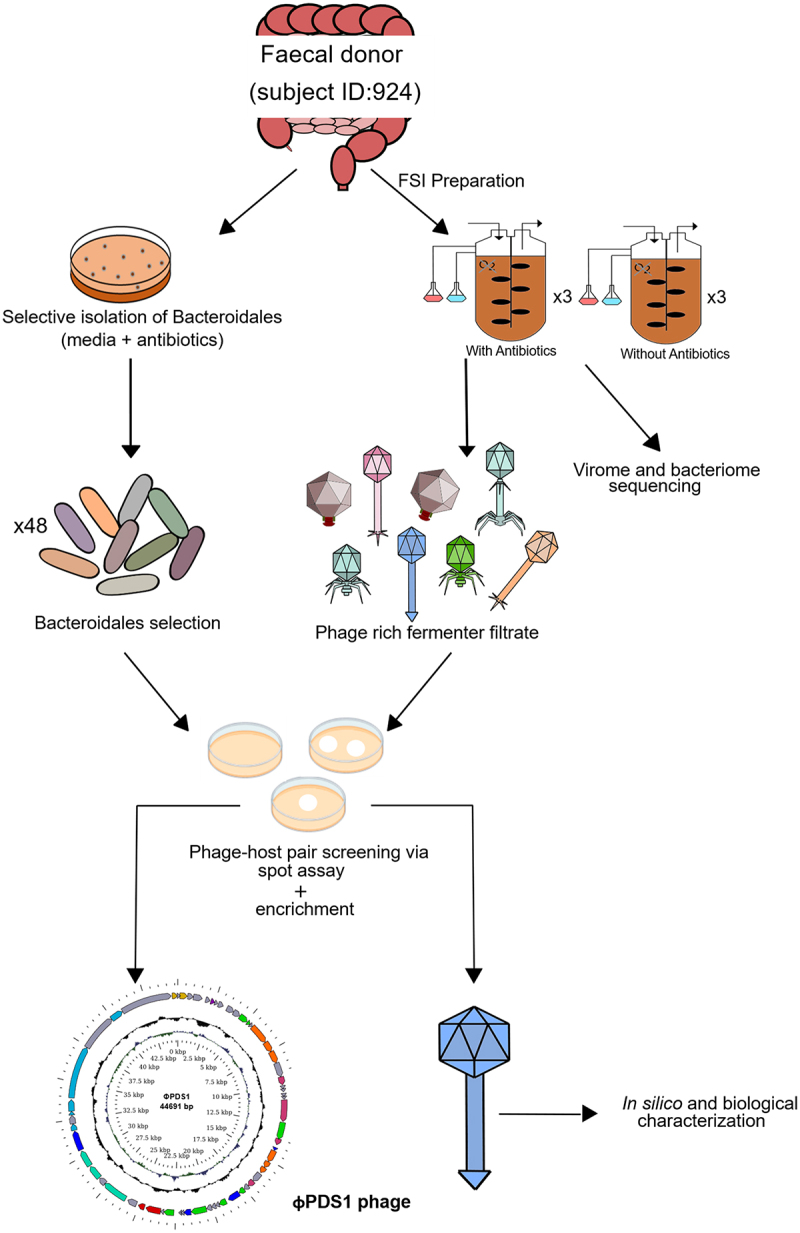
Schematic overview of the key experimental steps carried out to screen for novel phage-host pairs targeting Bacteroidales order bacteria.

A graphical overview of the steps taken to screen novel Bacteroidales phages is presented in Figure 1. A fecal sample from a healthy donor (subject ID: 924) was used to initiate batch fermentations, using two chemostat vessels in parallel, one of which contained vancomycin (7.5 μg/ml) and kanamycin (100 μg/ml) with the aim of inhibiting facultative anaerobes and gram-positive strict anaerobes, thus favoring the growth of strictly anaerobic gram-negative Bacteroidales. These fermentations were performed three times.

16S rRNA sequencing of total DNA extracted from the two vessels at different time points was already published in our previous work^23^ revealing a significant difference in relative abundance of bacterial genera (Figure S1). At T0 hours both vessels were dominated by the orders Clostridiales (~50%), Bacteroidales (~30%) including the genera *Parabacteroides* and *Bacteroides*, and Selenomonadales (~12.5%). By T4.5 hours, a significant change in the composition of bacterial communities was observed. In the vessel treated with antibiotics, the balance shifted toward the order Bacteroidales (~65%), while the vessel without antibiotics became dominated by gram-positive *Catenibacterium* (~75%). This observation supported the efficacy of vancomycin in inhibiting the growth of gram-positive anaerobic bacteria in our chemostat model. By T17.5 hours, the vessel treated with antibiotics showed a remarkable predominance of *Bacteroides* and *Parabacteroides*, representing approximately 90% of the relative abundance. In contrast, these genera contributed less than 20% of the relative abundance in the control vessel (without antibiotics). In the vessel without antibiotics an increase in *Faecalibacterium* was observed, accompanied by a reduction in the relative abundance of *Catenibacterium*. Subsequently, from T17.5 hours to T24 hours, no substantial shifts in the relative abundance of the genera were observed in either of the two vessels.

### 2.2. Identification of potential phage-host candidates targeting Bacteroidales order

In conjunction with the fecal fermentation, individual Bacteroidales isolates were obtained from the frozen standard inoculum (FSI) on various media (FAA, CBA, and YCFA-agar) containing the same antibiotics. To screen for novel phage-host pairs, the phage-enriched fermentation filtrate was screened against the isolated Bacteroidales species using spot-on lawn assays. A total of 48 colonies were selected based on differences in colony morphology. 16S rRNA gene sequencing was performed to obtain an initial estimate of the potential bacterial genus isolated and revealed that most of them belonged to the genus *Bacteroides*: *B. uniformis* (25 colonies), *B. dorei* (7), *B. fragilis* (1), *B. xylanisolvens* (1) and *B. ovatus* (1), and there was also a notable enrichment of *Parabacteroides distasonis* (13 colonies) (Table S1). These findings were consistent with the high prevalence of *Bacteroides* and *Parabacteroides* in the fecal fermentation with antibiotics (~90%). The T21-hour phage-rich fecal fermentate was spotted on lawns of the 48 isolated Bacteroidales species, resulting in consistent clearing specifically on the lawns of *P. distasonis* (Figure S2). Furthermore, the phage-rich fecal fermentate collected from the antibiotic vessel formed more readily visible spots during screening for phage-host pairs as compared to the non-antibiotic vessel, confirming that phages were also enriched using this approach (Figures S2B and S2C). These results show that the antibiotic selection facilitated the phage-host pair screening process.

### 2.3. Genome analysis of the novel isolated siphovirus φPDS1 and its host Parabacteroides distasonis APCS2/PD

**Figure 2.**
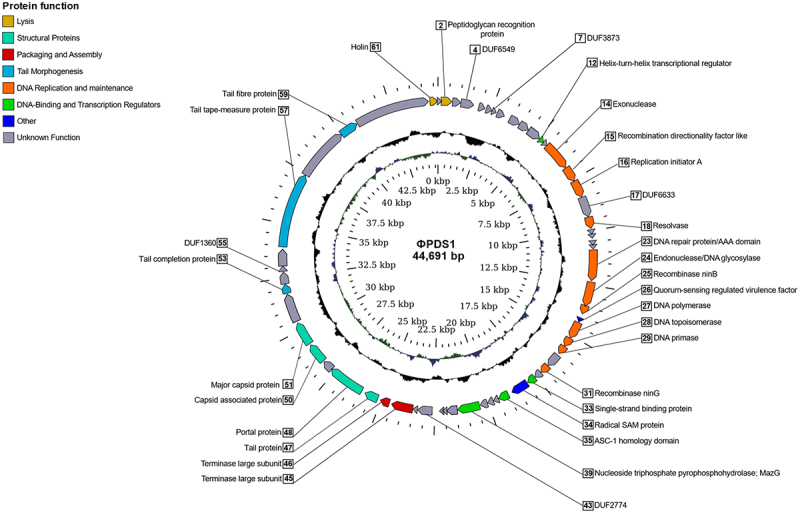
Circular genome map of the novel phage φPDS1 with a genome size of 44,691 bp. The innermost ring represents the GC skew (blue for the positive strand and green for the negative strand) while the central ring (black) displays the GC content. The outermost circle illustrates the coding genes (CDS) with HHpred predicted function as labels. The coloration of the CDS corresponds to their general functions as indicated in the legend. Genes whose function could not be determined are colored gray and remain unlabeled.

The phage that produced clearing areas on *P. distasonis*, designated as φPDS1, was subsequently propagated on its host, and subjected to shotgun genome sequencing. Reads were assembled into a circularized genome of 44,691 bp (GenBank MN929097; Figure 2). The GC content of the φPDS1 genome was determined to be 45.28 mol%, which closely matched the GC content of the host at 45.30 mol%. ORF prediction in the φPDS1 genome identified 61 putative protein-coding genes (CDS), and approximately half could be assigned functions using HHpred, VIGA and BLASTp (Figure 2, Table S2). These annotations included two structural head proteins, five tail-associated proteins, five DNA replication genes, three recombinase genes, two large terminase genes, and two lysis genes. Notably, the two large terminase genes represent the two domains of the large terminase split into two CDS (Figure S3). This observation raises the possibility of either a distinct codon table specific to the φPDS1 genome or a potential issue at the assembly level. The right-hand side of the genome (0−22.5 kbp) contained genes encoding functions associated with DNA replication and maintenance, while the left-hand side (22.5−44.7 kbp) was dominated by phage structural genes, along with genes associated with packaging and assembly. Lysogenic gene modules, which are typically composed of a serine or tyrosine integrase, a repressor of the lytic cycle and excisionase,^24^ were not identified in the φPDS1 genome.

The *Parabacteroides* genus was predicted as a potential host for φPDS1 with a score cutoff of 90% through the iPHoP analysis, which integrates different approaches to infer host genus of phages. *P. distasonis* and *P. sp900552465* were detected at the species level with higher confidence scores (92.9% and 91.1%, respectively). Notably, *P. sp900552465* represents an uncultured human gut *Parabacteroides* species (GenBank: GCA_900552465.1, Bioproject: PRJEB26432), derived from a metagenome-assembled genome isolated from human feces.^25^ Regarding the different methods applied, BLAST was concordant with these two species, while CRISPR-spacers targeting φPDS1 were found in *P. distasonis* and in *Odoribacter splanchnicus* (Table S3).

Whole genome sequencing of the φPDS1 host, designated as *Parabacteroides distasonis* APCS2/PD, revealed a single circular chromosome (GenBank CP042285) with a genome size of 5,344,828 bp (Figure S4A). Additionally, one associated circular plasmid, pPDS2–1, measuring 4,148 bp (GenBank CP042284), was identified (Figure S4B) and its annotation revealed that it carries genes encoding for a toxin-antitoxin system and vesicle mobilization. The host taxonomy was determined using the Genome Taxonomy Database (GTDB) and the associated taxonomic classification toolkit (GTDB-Tk), which classified the host as belonging to the *Parabacteroides* species with the closest phylogenetically related strain being *P. distasonis* DSM 20701, sharing an average nucleotide identity (ANI) of 97.32%.

### 2.4. φPDS1 is classified in a newly candidate genus, Sagittacolavirus, within the candidate family Paboviridae

**Figure 3.**
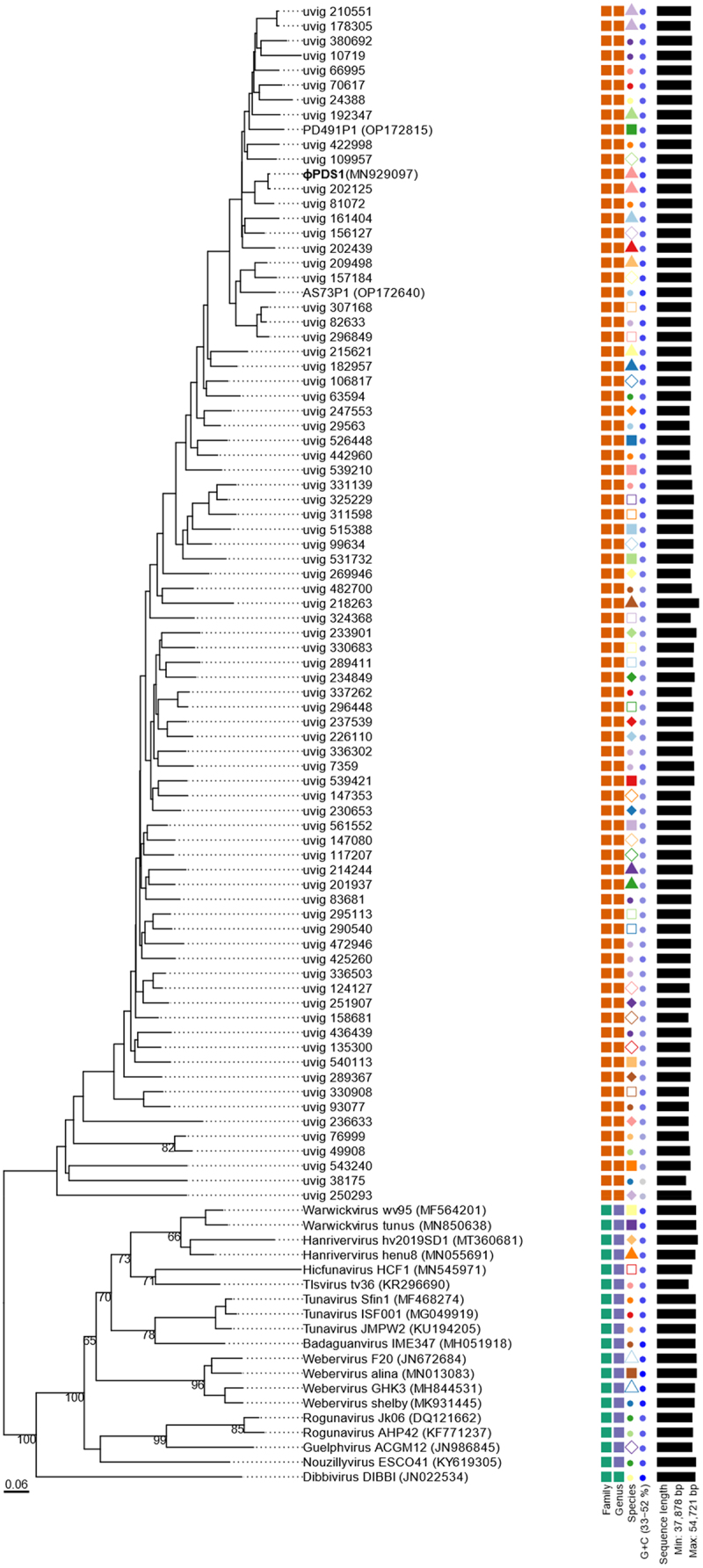
Phylogenomic genome BLAST distance phylogeny (GBDP) tree representing φPDS1, 78 members of the new candidate *Paboviridae* family, and using 19 phages from the *Drexlerviridae* family as outgroup. The tree was generated using the VICTOR tool with the D0 formula. The numbers above the branches indicate GBDP pseudo-bootstrap support values from 100 replicates. The branch lengths are scaled in terms of the respective distance formula used. Family, genus, species are tentatively grouped by their phylogenetic relationships when the same shape and color coincide. The GC content is represented in blue; the darker the color, the higher the content. Accession numbers are provided in brackets.

A new candidate family *Paboviridae*, within the class Caudoviricetes, has been recently proposed.^26^ This family infects bacteria belonging to the *Parabacteroides*, *Alistipes*, and other genera within the Bacteroidales order in the human gut. To determine whether φPDS1 could be classified within this candidate family *Paboviridae*, we followed a similar approach to Shen and colleagues.^26^ From the Gut Phage Database (GPD), we selected phage genomes with a completeness of at least 95% (as assigned by CheckV) for which we were able to detect the large terminase subunit (*n* = 313), along with the two experimental phages of their study, *Parabacteroides* phage PD491P1 and *Alistipes* phage AS73P1, to investigate phylogenetic relationships. Analyses conducted using VIRIDIC and VICTOR tools confirmed that φPDS1 belongs to the candidate family *Paboviridae* (Figure 3 and Table S4). We proceeded to perform a VIRIDIC analysis, based on intergenomic nucleotide similarities and which implements the traditional algorithm used by ICTV, to explore whether we could classify φPDS1 into a genus within this family. Out of the 316 total sequences analyzed, VIRIDIC detected a total of 38 genus clusters (Table S5). φPDS1 was grouped at the genus level (>70% similarity) with the PD491P1 phage and 58 other phages from the candidate family *Paboviridae*. Furthermore, it revealed a potential species cluster with uvig_202125 (97.01% similarity) (Table S5). We also inferred the phylogenetic tree using the VICTOR classifier. It grouped the representative phage genomes and φPDS1, within the same family (Figure 3). The VICTOR tree confirmed that φPDS1 belongs phylogenetically to the candidate *Paboviridae* family. However, the taxonomic annotation obtained for the genera of the outgroup phage genomes contradicted the ICTV genus annotation of the selected outgroup. We confirmed that the outgroup phage genomes were correctly clustered at the genus level using VIRIDIC. Therefore, we made the decision to disregard the genus annotation provided by VICTOR and exclusively relied on the annotation performed by VIRIDIC.

This leads us to propose the new candidate genus *Sagittacolavirus*, which includes the two isolated phages, φPDS1 and PD491P1, and 58 other phages from the candidate *Paboviridae* family (Table S6). Based on the CRISPR-spacer targeting results from Shen et al. (2023),^26^ these 58 phages, clustered within the new potential genus *Sagittacolavirus*, were found to target hosts in *Bacteroides* (47), *Parabacteroides* (9), and 2 unassigned genera, in addition to PD491P1 whose host is *Parabacteroides*. These phages exhibited a similar range of GC content, varying from 43.09 to 45.28 mol%, and had genome sizes ranging from 42,828 to 46,430 bp. Additionally, the percentage of similarity compared to φPDS1 ranged from 75.47% to 97.01%. The closest relative of φPDS1 was identified as uvig_202125 and the most distant uvig_70617.

**Figure 4.**
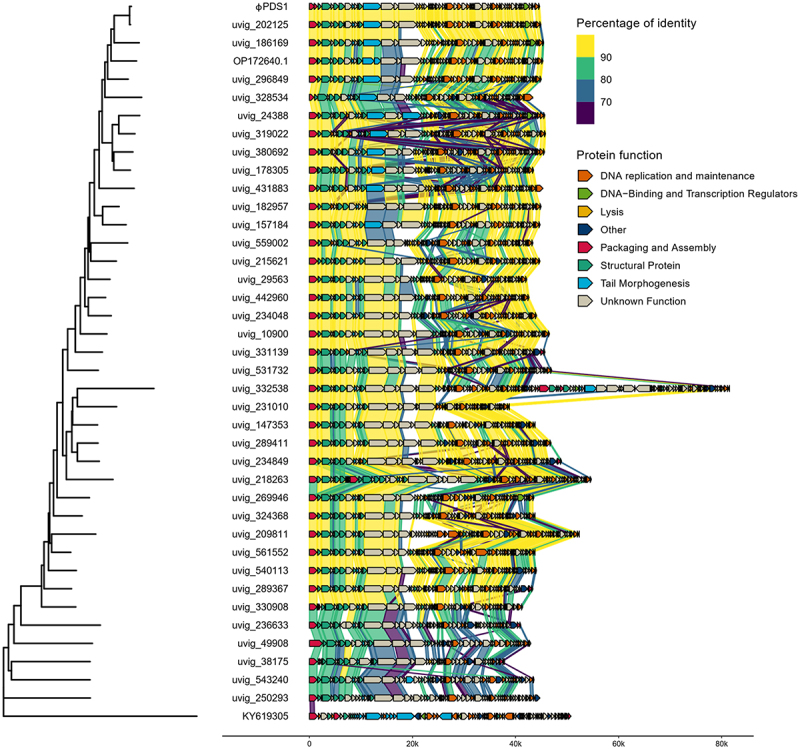
Whole genome comparisons of φPDS1 against members of the different genus clusters within the candidate *Paboviridae* family identified by VIRIDIC, along with one outgroup member from the *Drexlerviridae* family (KY619305; Nouzillyvirus ESCO41). The figure shows the percentage of identity of different proteins compared to the closest phylogenetic phage genome. The comparison was performed using Diamond. The color between two genes represents the percentage of identity between both at the protein level. The color of the gene is based on the annotation given by Pharokka. The left-side tree was inferred using the VICTOR tool and the figure was created using the R packages gggenomes and ggtree.

A member of each genus cluster (38) from the candidate *Paboviridae* family, together with φPDS1 and a member from an outgroup (KY619305; Nouzillyvirus ESCO41) were selected, and their genomes were aligned to observe the genetic synteny among them (Figures 4 and S5). Additionally, the intergenomic nucleotide similarities of these clusters were represented through VIRIDIC (Figure S6). The overall genome organization and genetic synteny were found to be largely conserved across all the clusters, except for the groups at the bottom of Figure 4, which showed lower percentages of identity. While most of the sequences showed a high percentage of identity between all the clusters, there was a region corresponding to a protein with an unknown function in which most of the clusters differed. The functions of the proteins encoded by the surrounding genes are related to tail morphogenesis, which suggests that it may have a related function. The member of the outgroup confirmed that there was no genomic similarity with these phages.

The prevalence of φPDS1 in the human gut was also studied based on the mapping of the φPDS1 genome to metagenomic reads from 965 human gut metagenomes with a coverage higher than 30% (Table S7). φPDS1 showed a prevalence of 31.4% in these samples, suggesting a high prevalence of this phage in the gut. The highest prevalence (59.6%) was observed in samples from Danish individuals (*n* = 109), while the lowest (22.6%) was noted in samples from Italian individuals (*n* = 62). No detected φPDS1 was determined in a Tanzanian cohort (*n* = 40).

### 2.5. Relative and absolute abundance of φPDS1 in the fecal fermenter

**Figure 5.**
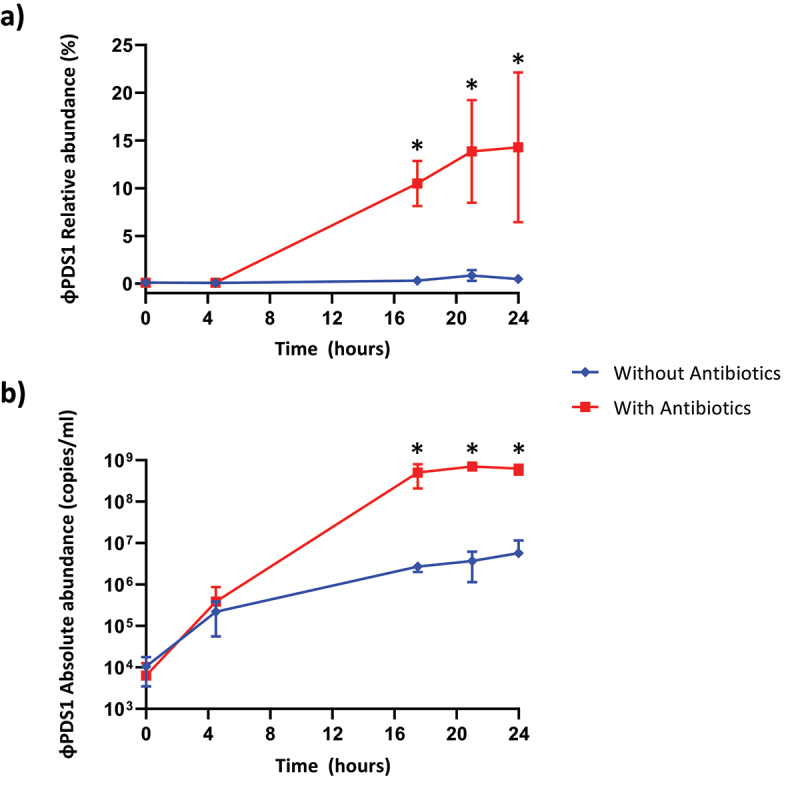
Abundance of φPDS1 in the fermenter vessel with and without selective conditions showing that the selective conditions greatly aided φPDS1 propagation. (a) Relative abundance of φPDS1 with and without antibiotics obtained from the total viral reads sequenced. (b) Absolute quantification of φPDS1 via qPCR targeting a segment of the phage DNA polymerase. Titre determined in copies/ml using the standard curve method. Error bars represent standard deviation (*n* = 3). * indicates statistically significant differences between vessels, with a *p*-value <0.05 after performing an unpaired *t*-test or the Mann – Whitney U test, depending on the data distribution (normal or non-normal, respectively).

The relative and absolute abundance of φPDS1 in the fermenters was determined under both conditions (with antibiotics vs no antibiotics). Relative abundance was obtained using the shotgun metaviromics dataset already published in our previous work,^23^ while the absolute abundance was measured by qPCR with the standard curve method. It was evident that the selective enrichment of the host greatly aided the propagation and expansion of φPDS1, which facilitated its isolation (Figure 5). The relative abundance of φPDS1, in relation to the total viral reads sequenced, showed that φPDS1 contributed to ~ 14% of the sequenced reads in the later time point samples collected from the antibiotic-treated vessel (Figure 5(a)). In contrast, less than 1% of reads belonged to φPDS1 in the untreated antibiotic vessel. Absolute quantification using qPCR also confirmed an increase in φPDS1 abundance over time. At the beginning of the fermentation (T0), the abundance of the phage was similar in both vessels (~1 × 10^4^ copies/ml). However, at the later time points the phage achieved a titer of ~ 6 × 10^8^ copies/ml in the antibiotic-treated vessel, while the titer in the non-treated antibiotic vessel was two logs lower at ~ 5 × 10^6^ copies/ml (Figure 5(b)). The results obtained for the whole virome, which were already published,^23^ support the φPDS1 selection via antibiotic treatment, as a reduction in virome diversity indices was documented in the presence of antibiotics.

### 2.6. Biological characterization of φPDS1

**Figure 6.**
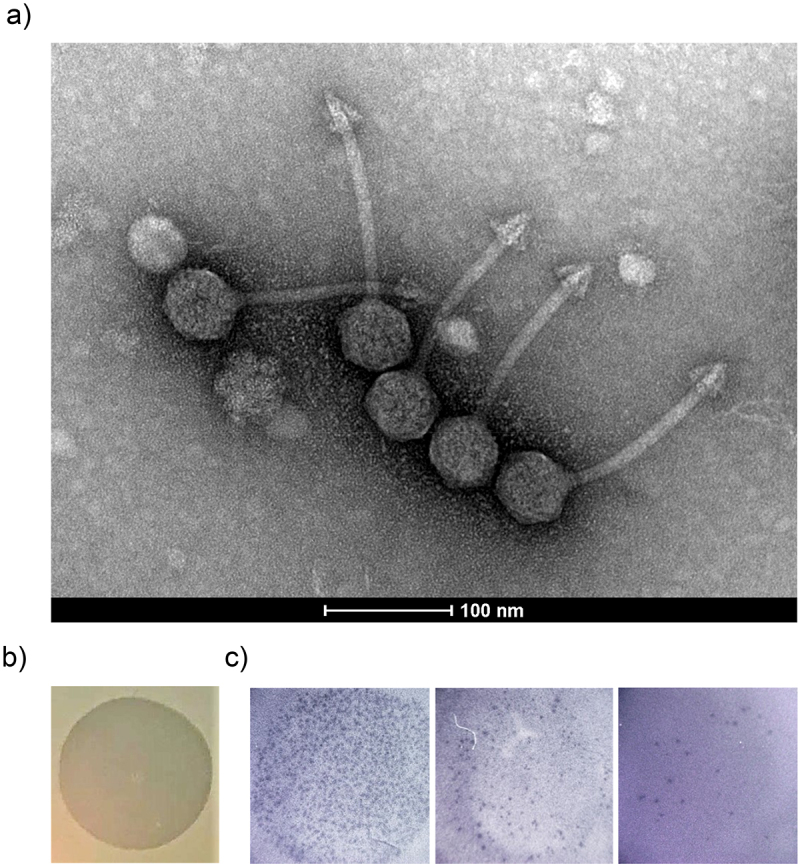
Characterisation and visualization of φPDS1 and formed spots and plaques. (a) Transmission electron micrograph of φPDS1 generated from the enriched lysate, stained with uranyl acetate showed that φPDS1 has a siphovirus morphology. The capsid diameter is approximately 53 ± 2.0 nm, and the tail length is 150 ± 10.0 nm. (b) Spot morphology with incomplete clearing. (c) Visualization of φPDS1 plaques from a spot assay at different titers using a stereoscopic microscope.

Transmission electron micrographs of the φPDS1-rich lysate showed that this phage has a characteristic siphovirus-like morphology with a long, non-contractile tail (Figure 6(a)). The arrow-shaped tail tip of the phage was unusual and larger than typically observed for other siphoviruses. The capsid diameter was approximately 53 ± 2.0 nm and tail length 150 ± 10.0 nm.

Spots on lawns produced by high titers of the phage were readily visible, but incomplete clearing was also observed (Figure 6(b)). Colonies within zones of incomplete clearing were picked and streaked up to five times. PCR analysis was performed to examine the presence of φPDS1, but all colonies tested were negative for the phage. φPDS1 also produces plaques on its sensitive host, but they are pinprick in size making visualization and enumeration challenging to the naked eye. Individual plaques after a spot assay were visualized through a stereoscopic microscope allowing for rough pfu enumeration (Figure 6(c)). However, qPCR allows for a more precise quantification.

**Figure 7.**
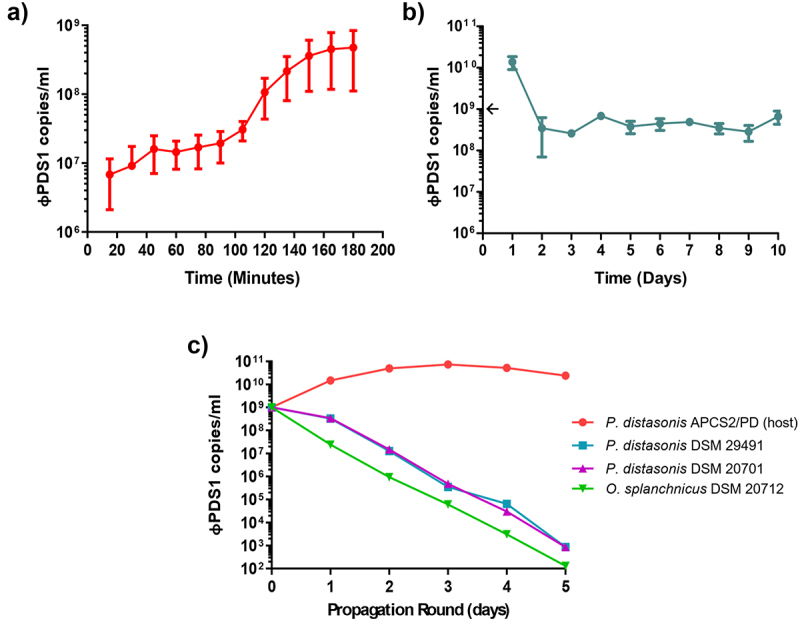
Biological characterization of φPDS1. (a) φPDS1 one-step growth curve. Sampling was performed every 15 minutes over 3 hours. The latent period was 90 minutes, and the burst size was ~ 23 copies per infected cell. (b) The titer of φPDS1 following continuous co-culture on the host over 10 days showing the persistence of the phage with its host. (c) Liquid propagation of φPDS1 on two commercial *P. distasonis* strains (DSM 29491 and DSM 20701) and *O. splanchnicus* (DSM 20712) was examined over five days with host *P. distasonis* APCS2/PD used as a control. Titres were determined in copies/ml via qPCR for all the experiments. Arrows on the y-axis indicate phage titer, after dilution, on initiation of the experiment. Error bars indicate standard deviation (*n* = 3).

A φPDS1 one-step growth curve was performed in triplicate with a MOI of ~ 1 (Figure 7(a)). The phage has a latent period of approximately 90 minutes with a relatively small burst size of ~ 23 copies per infected cell. Continuous co-culture of φPDS1 with its bacterial host showed that after 24 hours the phage titer recovered to ~ 1 × 10^10^ copies/ml following initial reductions consistent with dilution associated with sub-culturing (Figure 7(b)). By day two, the titer reduced to ~ 4 × 10^8^ copies/ml and remained at this approximate titer for subsequent rounds of sub-culturing (Figure 7(b)). This suggests that the presence of the phage selects for an increase in non-phage permissive host variants but does not render the culture fully resistant. The bacterial pellet collected at the end of the experiment was serially streaked three times. PCR confirmed that all colonies were φPDS1 negative.

φPDS1 showed stability within a temperature range of −80°C to 45°C, and a pH range of 3 to 11. Its activity decreased at 55°C and 65°C, resulting in the production of less clear spots on host lawns. The activity was completely lost at 80°C, and under extremely acidic (pH 1–2) and alkaline (pH 12–14) pH conditions.

We examined the ability of the φPDS1 to propagate on two *P. distasonis* strains obtained from a culture collection, DSM 29491 and DSM 20701, and an *O. splanchnicus* strain, DSM 20712, chosen following host-genus prediction analyses. The phage failed to propagate on the tested strains despite efficient propagation on *P. distasonis* APCS2/PD, as determined by qPCR (Figure 7(c)). The host range of φPDS1 was also examined using these strains, along with other strains from the Bacteroidales order (*P. goldsteinii* JCM13446, *B. intestinalis* APC919/174, *B. xylanisolvens* APCS1/XY and *B. thetaiotaomicron* VPI-5482), and no activity was observed against any of them.

The resistant mutation rate test found that  ~ 22% of *P. distasonis* APCS2/PD cells are resistant on initial exposure to φPDS1. This is consistent with lack of clearing of broth cultures and hazy spots on lawns. Additionally, we tested whether the percentage of resistant colonies was maintained when the bacteria had been in contact with the phage previously. The same experiment was carried out by plating *P. distasonis* APCS2/PD that had been co-cultured with φPDS1 for 48 hours. The results showed that the percentage of resistant colonies after being in contact with the phage for 48-hours increased by approximately ~ 22% to ~ 95%. This finding suggests that a phase variable mechanism may be involved in the persistence of the phage without impacting the number of bacteria.

In the absence of the phage, the host appeared to form two colony morphologies: larger round creamy colonies and smaller granular colonies. A greater count of the former morphology type was present on the phage-seeded plate, suggesting that possible phenotypic alterations have a role to play in morphology changes and the observed resistance. When φPDS1 was screened against lawns of these creamy colonies the zones of clearing were cloudier compared to spots that formed on lawns of *P. distasonis* APCS2/PD which was not recently exposed to the phage. The resistant colonies were re-streaked and colony PCR confirmed that all were φPDS1 negative.

## 3. Discussion

In recent years, many studies have revealed interesting insights into the human gut virome. However, most phages continue to exist only as sequence “dark matter”. Phages that have solely been characterized *in silico* can only provide limited information about their biological properties. Isolating and characterizing these phages is crucial for advancing our understanding of phage-host interactions in the human gut. The human gut virome is largely composed of dsDNA phages belonging to the recently established order *Crassvirales*,^12^ as well as ssDNA phages of the *Microviridae* family.^18,27,28^ However, the bacterial hosts for the majority of these phages remain unidentified. CRISPR-spacer analyses of virulent gut phages have indicated a notable association with hosts from the Clostridiales and Bacteroidales orders.^18^ To address this knowledge gap and to potentially shed light on phage-host interactions, the current study aimed to isolate novel human gut phages, with a specific focus on Bacteroidales due to the significant role and prevalence of this bacterial order within the human gut ecosystem. The majority of Bacteroidales species are classified within the genera *Bacteroides* and *Parabacteroides*, which are of significant importance and highly abundant in the human gut, where they have been associated with both health and disease.^16,17^ Although there have been multiple reports on phages infecting *Bacteroides*,^14,20,23,29–33^ only a limited number of studies have examined phages infecting *Parabacteroides*. Two *Parabacteroides*-associated prophages, ϕParabacteroides YZ-2015a and ϕParabacteroides YZ-2015b, were first detected in Sphagnum-dominated peat viromes. These prophages infect *P. distasonis* and *P. merdae*, respectively, and both belong to the ssDNA *Microviridae* family.^34^ Additionally, seven more *Parabacteroides* prophages of the *Microviridae* family were discovered in the gut of *Ciona robusta*, a marine vertebrate.^35^ Recently, Shen et al. (2023) have isolated 22 *Parabacteroides* phages from a collection of gut phage isolates.^26^ These phages were found to infect *P. faecis*, *P. distasonis*, and *P. merdae* and were isolated mainly from municipal sewage. Notably, one of these phages, PD491P1, showed a high prevalence in the human gut after screening 1,333 publicly available human gut metagenomic samples.^26^ This confirms the relevance of studying *Parabacteroides* phages in the human gut and highlights the limited knowledge currently available.

The selective enrichment method implemented in our study proved effective in facilitating the growth of Bacteroidales (Figure S1), resulting in the successful isolation of a novel lytic *P. distasonis* phage, φPDS1. This phage represents the first *P. distasonis*-targeting siphovirus to be isolated from human feces, biologically characterized, and deposited in NCBI taxonomy databases (NCBI:txid2709316). *P. distasonis* is a gram-negative, obligate anaerobe with rod-shaped morphology that is predominantly associated with the human gut.^36^ These bacteria have been recognized as important commensals with potential anti-inflammatory roles in the gut, contributing to the alleviation of metabolic disorders and obesity.^17,37–39^ Reduced abundance of the *Parabacteroides* genus has also been observed in the feces of patients suffering from IBD, as well as in patients with multiple sclerosis and rheumatoid arthritis.^40–43^ These findings could suggest potential for *P. distasonis* as a next-generation probiotic.^44^ However, several other studies have also linked *P. distasonis* to disease states, suggesting its potential role as an opportunistic pathogen.^17,22^ It has been reported that *P. distasonis* is the most abundant bacterial species in fecal samples collected from individuals suffering from Crohn’s disease (CD) and has been associated with enhanced colitis in DSS-induced mice.^45,46^ Furthermore, *P. distasonis* has been isolated from a lesion removed from the ileum of a CD patient.^47^ Considering the health and disease implications associated with this bacterial species, *P. distasonis* and φPDS1 should prove to be an intriguing phage-host pair for further study.

Annotation of the φPDS1 genome revealed that nearly half of the genes remain unidentified (Figure 2). However, among the successfully annotated genes, there were several with noteworthy functions. One of particular interest is gp_26, which encodes a quorum sensing (QS)-regulated transcription factor associated with virulence. QS is a bacterial phenomenon that allows cells to control the expression of specific genes in a population-density-dependent manner through cell-to-cell communication.^48^ This mechanism plays a crucial role in various processes such as virulence and biofilm formation. Furthermore, φPDS1 gene gp_34 encodes radical SAM enzymes, which are associated with the synthesis and metabolism of many cell compounds, and also they are involved in pathways that lead to the synthesis of the key players of QS molecules, known as autoinducers.^48^ Various QS response regulator homologs have been identified in sequenced phage genomes deposited in NCBI databases,^49^ and several examples of phages hijacking this process have been described. For instance, the *Vibrio cholerae* infecting phage, VP882, encodes a QS receptor homologous to that of its host. As a result, the phage can “listen in” on its hosts and monitor population densities thus allowing an informed lytic/lysogenic lifestyle switch. When the host cell density is high, the phage initiates lysis resulting in optimal propagation.^50^ Additionally, QS has been demonstrated to interfere with host phage defenses, aiding the infection process in a lytic *Pseudomonas aeruginosa* phage.^51^ Furthermore, φPDS1 encodes a single-stranded binding protein (SSB) which suggests that it does not rely on a host SSB for replication.^52^ Moreover, genes gp_25 and gp_31 encode Nin family recombinases, also known as *orf* and *rap*, which are associated with lytic growth.^53,54^ Another interesting feature is the presence of an auxiliary metabolic gene, MazG, encoded by gene gp_39. Such genes are of host origin and allow phages to alter host metabolic processes to their advantage. MazG genes have been mostly detected in marine phages. They are associated with both lytic and temperate lifestyles, and have been linked to maintenance of phage propagation in starved host cells by aiding metabolism.^55,56^ Based on the information presented, it is possible that φPDS1 possesses mechanisms that could potentially impact its host through the alteration of host metabolic processes and interference with host phage defenses. However, further studies are needed to accurately determine which mechanisms are truly involved in the interaction with its host.

Host-genus prediction analyses suggested that φPDS1 has evolved to infect *P. distasonis*. CRISPR spacers detected another potential member of the Bacteroidales order, *Odoribacter splanchnicus*. Some specific strains of *P. distasonis* (DSM 20701 and DSM 29491) and *O. splanchnicus* (DSM 20712) were obtained from DSMZ to test φPDS1 strain specificity. However, none of these strains were able to support the propagation of φPDS1 (Figure 7(c)), or produce clear spots on the lawns of these Bacteroidales strains. This suggests that φPDS1 is highly strain-specific in its infection strategy and has specialized to propagate on the *P. distasonis* APCS2/PD strain from which it was isolated.

A candidate phage family, *Paboviridae*, within the Caudoviricetes class, has been recently proposed.^26^ This family infects bacteria belonging to the *Parabacteroides*, *Alistipes*, and other genera within the Bacteroidales order in the human gut. Shen and colleagues identified this family based on the large terminase subunit of two isolated phages, PD491P1 and AS73P1, which infect *Parabacteroides* and *Alistipes*, respectively. Shen et al. included 258 complete phage genomes from the Gut Phage Database (GDP) in this candidate family. However, despite comparing the sequence identity of these two phages with φPDS1, they did not incorporate φPDS1 into their phylogenetic analysis. Our phylogenetic analysis has shown that φPDS1 could also be included in the candidate family *Paboviridae*, and we propose the formation of a new candidate genus, *Sagittacolavirus*, within this family after performing a VIRIDIC analysis. This genus includes φPDS1, PD491P1, and other 58 phage complete genomes from the GDP. While they found a 97.37% sequence identity by BLASTn between φPDS1 and PD491P1, our VIRIDIC analysis based on intergenomic similarities, revealed 78.5%, suggesting they could be clustered within the same genus but not at the species level. Regarding phage morphology, φPDS1 and PD491P1 are very similar, both exhibiting the characteristic arrowhead-like tail tip. This suggests that this feature may be a distinctive characteristic of this phage genus. Shen and colleagues described a similarity percentage of 82.82% between *Alistipes* phage AS73P1 and φPDS1 using BLASTn.^26^ However, our VIRIDIC results showed 66.83% of similarity, which prevented its inclusion within the proposed genus *Sagittacolavirus*. This difference could be related to the distinct host genera target (*Alistipes* and *Parabacteroides*), and differences in phage morphology, since although both are long-tailed, it appears that AS73P1 does not possess the characteristic arrowhead-like tail tip. The prevalence of φPDS1 in the human gut (31.4%) was shown to be higher than that of PD491P1 (29.5%) and AS73P1 (19.5%)^26^ after performing the same analysis. The only difference was that the number of human gut metagenomes used to map our phage, which was slightly lower (965 vs 1,333). This result supports the notion that this family of phages appears to be very prevalent in the human gut.^26^

φPDS1 failed to clear liquid cultures of their host despite efficient propagation, and zones of clearing remained slightly hazy. The phage was also unable to form stable lysogens, and no integrase was detected in its genome, strongly indicating that the phage is lytic. φPDS1 also demonstrated the ability to stably coexist with its host during serial co-culturing. Interestingly, previous studies have observed similar behavior in apparently lytic phages, where they fail to clear the liquid culture of their hosts. For instance, the lytic phage φcrAss002, persists and attains high titers but lacks the ability to form plaques or clear liquid cultures of its host *B. xylanisolvens*.^23^ More similar to φPDS1, the *B. intestinalis* phage, φcrAss001,^15,20^ and *B. thetaiotaomicron* phages^14,57^ can form large plaques on their hosts and propagate at high titers, but also do not produce clear liquid cultures. This phenomenon could be explained by the phase-variable expression of host capsule polysaccharide loci and surface features, which create transient phenotypic heterogeneity within an isogenic population.^15^ This heterogeneity results in a mixture of phage-permissive and non-permissive host variants, thus allowing both the phage and host to coexist.^14,15^ This could explain the observed difference in the percentage of resistant cells before (~22%) and after a 48-h co-culture with φPDS1 (~95%). A potential phase variable mechanism may be promoting the growth of bacterial subpopulations that are resistant, leading to a reduction in the sensitive subpopulations. However, this level of resistance may be sufficient for φPDS1 and its host to coexist. Phase-variable regions have been previously reported among gut *P. distasonis* strains.^58–60^ Phase variation of surface structures involves the reversible inversion of DNA regions containing promoters, resulting in the expression or suppression of downstream genes depending on the orientation.^61^ Invertible promotor regions are characteristic of Bacteroidales species residing in the human gut and are generally not conserved among Bacteroidales occupying other niches.^59,61^ This highlights the importance of host factors in influencing phage-host interactions. Further investigation, such as transcriptomics analysis, may provide insights into the role of these features and help understand the mechanisms mediating the interaction between φPDS1 and *P. distasonis* APCS2/PD.

In conclusion, we report the isolation, biological and *in silico* characterization of φPDS1. This phage is the first characterized lytic siphovirus to be isolated from the human gut that infects *P. distasonis*, and it has been deposited in the NCBI Taxonomy database, making it the first of its type to be documented. Additionally, we propose that φPDS1 may be classified in the new candidate genus *Sagittacolavirus* within the candidate family *Paboviridae* and the class Caudoviricetes. Considering the health and disease associations of *P. distasonis*, this phage-host pair merits further investigation which may also provide interesting insights into phage-host interactions in the human gut.

## 4. Material and methods

### 4.1. Donor recruitment and sample collection

A healthy female donor in her forties, designated as subject ID: 924, was recruited for fecal sample donation in accordance with the study protocol APC055 and ethics approved by Cork Research Ethics Committee. This individual had been previously identified as a persistent carrier of crAss-like phages, which infect commensal bacteria of the order Bacteroidales.^18,23,62^ Given these findings, the subject was deemed a potential donor for the *in vitro* isolation of potential novel phages that infect hosts of the Bacteroidales order.

### 4.2. Fecal fermentation with selective antibiotic enrichment

The fecal sample was processed immediately upon receipt into frozen standard inoculum (FSI) as described elsewhere.^23^ Fermentation medium was prepared as detailed in Guerin et al. (2018).^62^ The FSI was aliquoted into three volumes to perform the fermentations in triplicate, and they were run in batch format over 24 hours at 37°C following the same conditions outlined in Guerin et al. (2021).^23^ Two fermenter vessels were established in parallel, one with the addition of antibiotics (7.5 μg/ml vancomycin (Sigma-Aldrich, MO, USA) and 100 μg/ml kanamycin (Sigma-Aldrich, MO, USA)) to the YCFA-GSCM broth post-autoclaving, while the other served as a control without the incorporation of antibiotics. The antibiotics were chosen to selectively promote the growth of Bacteroidales via the elimination of gram-positive bacteria (vancomycin) as well as to limit faster growing facultative anaerobes (kanamycin).^23^ Samples were collected at specific time points: 0, 4.5, 17.5, 21, and 24 hours and were directly processed through centrifugation at 4,700 rpm at 4°C for 10 minutes. The resulting supernatants were filtered through a 0.45 μm pore polyethersulfone (PES) membrane filter (Sarstedt, Nümbrecht, Germany) and were stored at 4°C. The remaining bacterial-rich pellets were stored at −80°C.

### 4.3. Extraction of total DNA, 16S rRNA gene sequencing library preparation, and analysis of 16S rRNA gene sequencing data

Fecal pellets generated following centrifugation of fermentation samples were used to extract total DNA as described elsewhere.^23^ PCR amplification of hypervariable regions V3-V4 of bacterial 16S rRNA gene and sequencing library preparation were carried out following the procedure outlined in Shkoporov et al. (2018).^63^ FastQC (v0.11.5) was utilized to visualize the quality of the raw reads. Trimmomatic (v0.36) was employed to perform quality filtering on the reads.^64^ The filtered reads were imported into R (v3.4.3) and subjected to error analysis with the DADA2 package (v1.6.0).^65^ Detected errors were corrected through additional quality filtering and trimming, resulting in the generation of unique Ribosomal Variant Sequences (RSVs). Chimera filtering was performed on the RSVs using both the *de novo* and reference-based chimera filtering implemented in USEARCH (v8.1.1861) with the ChimeraSlayer gold database (v20110519).^66^ The remaining RSVs were classified with mothur (v1.34.4)^67^ against the RDP database (v11.4), as well as classified with SPINGO to species level.^68^ The resulting RSVs were further analyzed to assess the relative abundance of bacterial genera within the antibiotic- and non-antibiotic-containing vessels. R package ggplot2 (v2.2.1) was used to visualize the abundance data.

### 4.4. Novel phage-host pair screening

To selectively isolate anaerobic Bacteroidales species, ten-fold serial dilutions of the FSI were prepared using fresh Fastidious Anaerobe Broth (FAB) (Neogen, MI, USA). 100 μl of each was spread plated onto plates with different media containing vancomycin (7.5 µg/ml) and kanamycin (100 µg/ml). The media used were: Fastidious Anaerobe Agar (FAA) (Neogen, MI, USA), YCFA-Agar, and Columbia Blood Agar (CBA) (Oxoid, Hampshire, UK) with 5% sheep blood (TCS Biosciences, Birmingham, UK) supplemented with 25 μg/ml hemin (Sigma-Aldrich, MO, USA) and 100 μg/ml vitamin K (Sigma-Aldrich, MO, USA). The dilution plates were incubated anaerobically at 37°C for 48 hours. Colonies were restreaked, and the isolated colonies were incubated both aerobically and anaerobically to ensure the absence of aerobes. An estimation of species identification was carried out via Sanger sequencing of the 16S rRNA region using the universal bacterial primers 27F primer 5′-AGAGTTTGATCCTGGCTCAG-3′ and 1492 R primer 5′-GGTTACCTTGTTACGACTT-3′. Samples were prepared following the guidance for the LightRun Tube service (GATC Biotech AG, Ebersberg, Germany). BLASTn analysis was conducted on the obtained sequences against the NCBI 16S ribosomal RNA sequences (Bacteria and Archaea) database using standard parameters.

48 pure cultures of Bacteroidales were used in the phage-host pair screening. Overnight cultures were prepared in 10 ml of FAB and an agar overlay method was used for screening. From each of these cultures, 300 µl was added to 0.3% FAA agar overlay (0.3% agar w/v), with MgSO_4_ and CaCl_2_ (1 mM final concentration). This mixture was poured onto pre-prepared FAA base agar (1.5% agar w/v), and 5 µl of the filtered fermentation supernatants collected from the antibiotic and non-antibiotic containing vessels at 21 h were spotted onto the lawns of each culture and dried. The original fecal filtrate, prepared from subject ID: 924 feces, was also spotted on the lawns. The plates were incubated anaerobically at 37°C for 48 hours. Formed spots were picked using an inoculation loop, placed into 100 µl of SM buffer (1 M Tris HCl pH 7.5, 5 M NaCl, 1 M MgSO_4_), vortexed and incubated at room temperature for 5 hours. The resuspended spots were spun in a desktop centrifuge at maximum speed for 10 minutes, and the supernatant was filtered through a 0.45 μm pore PES membrane filter. Ten-fold serial dilutions of the lysates were prepared in SM buffer. Spot assays were then repeated using each lysate on lawns of the culture on which it was originally spotted on. Plaque assays were also performed using 3 ml of 0.3% FAA agar (0.3% agar w/v), with the addition of MgSO_4_ and CaCl_2_ (1 mM final concentration), 300 µl of each overnight culture on which spots formed and 50 µl of phage dilution. Following this, spot picking and spot assays were repeated a third time. Spot formation was consistently observed in all cultures identified as *Parabacteroides distasonis* through Sanger sequencing of the 16S rRNA region. Four *P. distasonis* cultures, on which spot formation was least cloudy, were chosen for phage enrichment. These cultures were designated as FAA-S8, FAA-B5, FAA-B8, and CBA-S2 (Table S1).

### 4.5. Novel phage enrichment and shotgun sequencing of the phage and fermenter virome

Six rounds of serial enrichment of the phage were performed over six days on the *P. distasonis* cultures to increase the titer. Each round of propagation was performed using *P. distasonis* at OD_600_ = ~0.2 which was reached approximately 5 to 6 hours post sub-culturing of 100 μl of overnight culture in 10 ml of fresh FAB plus MgSO_4_ and CaCl_2_ (1 mM final concentration) with anaerobic incubation at 37°C. Once the culture was at the desired optical density, 1 ml of phage lysate was added at an unknown titer to the culture and incubated anaerobically overnight at 37°C. The propagations were centrifuged at 4,700 rpm at 4°C for 10 minutes and passed once through a 0.45 μm PES membrane syringe filter. Filtrates were then stored at 4°C. Each round of propagation was performed following the same procedure with 1 ml of the prior phage lysate added to *P. distasonis* culture at OD_600_ = ~0.2. To ensure maintenance of phage throughout the serial propagations, spot assays were performed as described above. On completion of the enrichment, spot and plaque assays were repeated to check for improved clearing.

Nucleic acids were extracted from 10 ml of filtered phage lysate generated from the final round of enrichment on each of the *P. distasonis* cultures. The virus-like particle (VLP) purification and DNA extraction steps were performed as described elsewhere.^63^ The extracted DNA was then purified using the DNeasy Blood & Tissue Kit according to the manufacturer’s instructions with a final elution volume of 50 μl. The DNA was quantified using Qubit dsDNA HS Assay kit (ThermoFisher Scientific, Vilnius, Lithuania) and was directly subjected to random shotgun library preparation using Nextera XT DNA Library Preparation Kit (Illumina) without preliminary multiple displacement amplification (MDA). Normalization was performed according to the manufacturer’s protocol using the bead-based method. The prepared libraries were sequenced using 2 × 300bp paired-end chemistry on an Illumina MiSeq platform (Illumina, CA, USA) at GATC Biotech AG (Ebersberg, Germany). The quality of the raw reads was analyzed using FastQC (v0.11.5). Removal of Nextera adaptors was performed with Trimmomatic (v0.36)^64^ with the following parameters: minimum length of 60, a sliding window size of 4 and a minimum Phread score of 33. The trimmed and filtered reads were then assembled into contigs using SPAdes (v1.13.1).^69^

Additionally, extraction of VLPs after enrichment from 10 ml of collected filtered fermentation supernatants was carried out, following the established protocol described by Shkoporov et al. (2018).^63^ Sequencing of the fermenter virome and its analysis are described in Guerin et al. (2021).^23^ The relative abundance of the reads generated following sequencing of the fermenter samples were aligned to the novel phage. Results were expressed as novel phage reads relative to the total number of reads.

### 4.6. In silico analyses and characterisation of φPDS1

Annotation of the novel phage genome, denoted as φPDS1, was performed using *de novo* viral genome annotator VIGA.^70^ Manual functional analyses were performed on the predicted protein-coding sequences using HHPred and BLASTp to generate a more detailed annotation. HHPred annotations were performed with the following databases: PDB_mm_CIF70_18_Jun, Pfam-A_v35, NCBI_CD_v3.19, and TIGRFAMs_v15.0.^71^ A genomic map of the φPDS1 genome was generated using the Proksee tool with annotations incorporated.^72^

The genome sequence of φPDS1 was compared with 506 phage genomes from the Gut Phage Database (GPD) of the candidate *Paboviridae* family. This family is characterized as infecting *Parabacteroides*, *Alistipes*, and other bacterial genera within Bacteroidales order in the human gut.^26^ CheckV (v1.0.1) was used to ensure the completeness of all the genomes.^73^ The genomes with a completion of at least 95% were selected to minimize bias in the subsequent genomic analysis, resulting in a total of 313 complete phage genomes. VIRIDIC^74^ was used to calculate virus intergenomic similarities of all complete phage genomes (*n* = 313) belonging to the candidate family *Paboviridae* obtained from GPD. The number of complete genomes exceeded the maximum limit allowed in the VICTOR classifier, which is 100 genomes. To address this, we decided to reduce the number of phages for comparison while preserving the maximum diversity in the sample. To achieve this, large terminase genes were identified using Pharokka (v1.3.2).^75^ Subsequently, the 313 terminases were aligned using MAFFT (v7.520) (linsi algorithm, using the options – dash and – originalseqonly).^76^ The protein alignment was trimmed using clipkit (v1.3.0) (options, -m kpic).^77^ Maximum likelihood trees were then inferred from the curated alignment using IQ-TREE (v2.2.0.3) (options -nm 4,000).^78^ Node support values were assessed using the options -bb 1,000 for ultrafast bootstraps and -alrt 1,000 for SH-aLRT. The best evolutionary model was selected using ModelFinder (BIC criterion).^79^ The resulting tree was used to select the 80 most diverse genomes (representative genome) with Treemmer (v0.3).^80^ These representative genomes, along with φPDS1 and 19 *Drexlerviridae* phages (used as an outgroup), were compared using the VICTOR classifier^81^ to investigate whether φPDS1 belongs to the candidate family *Paboviridae*, resulting in the construction of a phylogenetic tree inferred by the Genome-BLAST Distance Phylogeny method with default parameters and D0 formula. A sequence from each of the genus-level clusters obtained in the VIRIDIC analysis was selected as a representative to perform a pairwise comparison, in order to examine synteny and relatedness among these phages and φPDS1. iPHoP tool was used for computational prediction of the host genus of φPDS1 based on its genome sequence with a score cutoff of 90%.^82^ This tool infers candidate phage-host pairs integrating results from multiple host prediction approaches (blast, CRISPR, WIsH, VHM, PHP and RaFAH).^82^

To compare the genomic organization of the different phages within the genera defined by VIRIDIC, we randomly selected one genome per genus as a representative of the cluster, and we added one phage as the representative of the outgroup. All phages were annotated using Pharokka v1.3.2.^75^ Specifically, coding sequences (CDS) were predicted using PHANOTATE. Functional annotation was generated by matching each CDS to the PHROGs, VFDB, and CARD databases using MMseqs2 and PyHMMER. The different genomes were compared with each other using BLASTn (v2.14.0) (options -task blastn, -evalue 1, -max_target_seqs 25,000) for nucleotide full genome comparison and Diamond v2.1.8 (options blastp, -k0, –ultra-sensitive – algo 1) for protein comparison. A phylogenetic tree was inferred using the reference genomes with the VICTOR classifier,^81^ to arrange the genomes by order of phylogenetic proximity. The comparison figure was generated using the R packages gggenomes and ggtree.^83^

The prevalence of φPDS1 in the human gut was analyzed by mapping the φPDS1 genome to metagenomic reads from 965 human gut samples from five different publicly available studies (PRJEB5224, PRJEB6997, PRJNA422434, PRJNA392180, PRJNA553191)^84–88^ using BBmap^89^ with default parameters. A φPDS1 genome with a coverage of more than 30% was considered to be present in the metagenomic samples. Accession numbers for the sequence read archives and information related to the human gut metagenomic samples are detailed in Table S7.

The host’s complete genome was used to determine its taxonomy using the Genome Taxonomy Database (GTDB) (v214.1) and the associated taxonomic classification toolkit (GTDB-Tk) (v2.3.0) with an average nucleotide identity (ANI) radius > 95%.^90^

### 4.7. Transmission electron microscopy

A 50 ml pool of φPDS1 lysate prepared from the serial enrichment was concentrated via ultra-centrifugation using a F65L-6×13.5 rotor (Thermo Scientific, MA, USA) at 120,000 g for a total of 3 hours at 4°C. Finally, the pellets were resuspended in a final volume of 5 ml of SM buffer. The suspensions were then applied to a step gradient of 5 M and 3 M cesium chloride (CsCl) solutions followed by centrifugation at 105,000 g for 2.5 hours at 4°C. The band containing viral particles was collected and subjected to CsCl clean-up steps.^62^ A spot assay was performed to confirm the presence and titer of φPDS1 in the concentrated purified fraction. Five microliter aliquots of the concentrated viral fraction were applied to Formvar/Carbon 200 Mesh, Cu grids (Electron Microscopy Sciences, PA, USA) with subsequent removal of excess sample by blotting. Grids were then negatively contrasted with 0.5% (w/v) uranyl acetate and examined at UCD Conway Imaging Core Facility (University College Dublin, Dublin, Ireland) by Tecnai G2 12 BioTWIN transmission electron microscope.

### 4.8. Quantitative real-time PCR (qPCR), primer and standard development and quantification of φPDS1

The φPDS1 major capsid protein was chosen for qPCR primer and standard development. Primers (Fwd 5‘GGAACAACGGGACGATTG-3’ and Rev 5’-CAATCACGGACGCAATAGG-3’) were designed using PERL Primer software and CLC Sequence Viewer 8.0. These primers detect φPDS1 producing a 193 bp PCR product with the following conditions: initial denaturation at 95°C for 5 minutes, then 35 cycles of 95°C for 20 seconds, 60°C for 20 seconds, 72°C for 20 seconds and a final cycle at 72°C for 1 minute. To develop standards for qPCR calibration curves, products from these primers were cloned into pCR2.1-TOPO TA vector (ThermoFisher Scientific, Vilnius, Lithuania). Extracted plasmids were quantified using Qubit dsDNA BR Assay kit (ThermoFisher Scientific, Vilnius, Lithuania) and diluted to 10^9^ copies/μl based on molar mass of DNA. Ten-fold serial dilutions of the plasmids were used to build a standard calibration curve. To quantify φPDS1 in the fecal fermenters, qPCR of viral nucleic acids from filtered fermentation supernatants was performed at all time points in triplicate. This was carried out in a 15 μl reaction volume using SensiFAST SYBR No-ROX mastermix (Bioline, London, UK) in a LightCycler 480 thermocycler (Roche) with the following conditions: initial denaturation at 95°C for 5 minutes, then 45 cycles of 95°C for 20 seconds, 60°C for 20 seconds and 72°C for 20 seconds. Resulting Ct-values were converted to copies/ml based on the generated calibration curves.

### 4.9. Shotgun sequencing of P. distasonis APCS2/PD using Illumina and Oxford Nanopore platforms

Bacterial genomic DNA was extracted from 10 ml overnight cultures of *P. distasonis* APCS2/PD using phenol/chloroform with precipitation in 3 M sodium acetate and cold absolute ethanol for the generation of long- and short-read sequences for hybrid assembly. DNA was quantified using Qubit BR DNA Assay Kit. Short-read shotgun sequencing of the extracted DNA was carried out using the Accel-NGS 1S Plus DNA Library Kit (Swift Biosciences, MI, USA) and Illumina HiSeq 4000 technology following manufacturer’s instructions. Long-read Oxford Nanopore library preparation was performed according to the manufacturer protocol for Rapid Barcoding Sequencing Kit (SQK-RPK004; Oxford Nanopore Technologies, UK) with the adaptions described by Guerin et al., (2021).^23^ Pooled samples were loaded into SpotON Flow Cell (Oxford Nanopore Technologies, UK) and MinION sequenced for 48 hours (Oxford Nanopore Technologies,UK). Hybrid assembly of quality-filtered and trimmed Illumina and raw Nanopore reads was conducted by hybridSPAdes (v1.13.1).^91^ Nine scaffolds greater than 1 kb were generated and were manually curated, joined, and circularized using CLC Sequence Viewer. The assembled and circularized genome and associated plasmid were then submitted to NCBI Prokaryotic Genome Annotation Pipeline. The GenBank file for the genome and associated plasmid was visualized using Proksee.^72^

### 4.10. Biological characterization of φPDS1

A one-step growth curve was performed in triplicate to determine the latent period and burst size of φPDS1. An early logarithmic phase culture of *P. distasonis* APCS2/PD was infected with φPDS1 with a multiplicity of infection (MOI) of ~ 1. Following incubation at room temperature for 5 minutes, the infected culture was centrifuged at 5,000 rpm for 15 minutes at 20°C, and the pellet was resuspended with FAB. The phage-host pair were maintained at 37°C under anaerobic conditions for 3 hours with 1 ml sample collection every 15 minutes. Samples were centrifuged and the supernatants were filtered through 0.45 µM pore syringe filters. Analysis was performed using absolute qPCR with the standard curve method and primers as described above.

The calculation of the resistant mutation rate was carried out by pouring 3 ml of 0.3% FAA molten overlay containing different 10-fold serial dilutions of an overnight culture of *P. distasonis* APCS2/PD (100 µl), along with 100 µl of high-titer φPDS1 lysate (>10^10^ copies/ml) on FAA agar plates. Negative controls were prepared in the same manner, excluding phage. The plates were incubated anaerobically for 48 hours at 37°C. The resistant mutation rate was calculated as the percentage of colonies on the phage-containing plate versus counts for the equivalent negative control. To assess the maintenance after the cohabitation of *P. distasonis* APCS2/PD with φPDS1, the same experiment was conducted plating *P. distasonis* APCS2/PD that had been co-cultured with φPDS1 for 48 hours. Colony morphology was observed, and sixteen colonies were picked and restreaked four times. Standard PCR was performed using primers specific to φPDS1 as described above to check for the potential integration of the phage into the host genome. Spot assays were always performed to check for host resistance after phage exposure.

The thermolability and pH tolerance of φPDS1 were examined after treating it at different temperatures (−80°C, −20°C, 4°C, 25°C, 37°C, 45°C, 55°C, 65°C, and 80°C) and pH (1–14) ranges for 24 hours. The determination of the phage’s stability was based on its ability to produce spots using the overlay method after the treatments.

Co-culturing of φPDS1 and *P. distasonis* APCS2/PD was performed by serial sub-culturing over ten days in triplicate. This was initiated using 1 ml of φPDS1 lysate at 10^10^ copies/ml that was introduced into 10 ml of *P. distasonis* APCS2/PD culture in FAB at OD_600_ = 0.2. Subsequent rounds of sub-culturing were performed by introducing the prior co-culture into 10 ml fresh FAB at a ratio of 1:50. φPDS1 titer was quantified using qPCR. The bacterial pellet collected, following generation of the final phage lysate on day ten, was serially streaked three times, and the resultant colonies were examined for the presence of the phage by standard PCR.

The ability of the φPDS1 to infect other commercially available *P. distasonis* strains, DSM 29491 and DSM 20701 (ATCC 8503), and *Odoribacter splanchnicus* DSM 20712 was investigated via liquid propagation using the same method as described above for phage enrichment. Propagation of φPDS1 on *P. distasonis* APCS2/PD was performed in parallel as a control. Propagation of the phage on the other bacterial strains was examined via absolute qPCR as described above. The host range of φPDS1 was also tested by spotting serial dilutions of the phage suspension onto FAA overlays seeded with these bacterial strains. The host range test also included other strains from the Bacteroidales order: *P. goldsteinii* JCM13446 (EU136697), *B. intestinalis* APC919/174 (CP041379), *B. xylanisolvens* APCS1/XY (CP042282) and *B. thetaiotaomicron* VPI-5482 (CP092641).

## Supplementary Material

Supplemental Material

Supplemental Material

TableS3.xlsx

TableS1.xlsx

TableS7.xlsx

TableS2.xlsx

TableS4.xlsx

SupplementaryMaterial_PDS1_submission_clean.docx

## Data Availability

All data generated or analyzed during this study are included in this report and in the supplementary material. The genome of φPDS1 is deposited into GenBank under accession MN929097 (assembled and annotated genome). The genome of the bacterial host of φPDS1, *Parabacteroides distasonis* APCS2/PD, is deposited under the following accession codes: BioProject PRJNA556872, GenBank CP042285 (assembled and annotated genome) and associated plasmid pPDS2–1, GenBank CP042284.
