# Supplementary material for: Isolation and characterization of a novel lytic Parabacteroides distasonis bacteriophage φPDS1 from the human gut
Source: Gut Microbes. 2024 Jan 4;16(1):2298254. doi: 10.1080/19490976.2023.2298254 (PMC10773633; doi:10.1080/19490976.2023.2298254)
Supplement: SupplementaryMaterial_PDS1_submission_clean.docx [file KGMI_A_2298254_SM5106.docx]

**Supplementary material**

**Table S1.** Identified bacterial species through Sanger sequencing of the 16S rRNA region of bacteria enriched from the faecal fermentation following antibiotic enrichment. *P. distasonis* species are highlighted in bold. CBA: Columbia blood agar; FAA: fastidious anaerobic agar; vitK: vitamin K; YCFA: yeast extract, casitone, fatty acids agar.

**Table S2.** Functional annotation of φPDS1 genome determined by HHpred, VIGA and BLASTp considering a cut-off probability higher than 90% and E value < 0.05. Prob: Probability.

**Table S3.** Top three hits obtained from each method used by the iPHoP tool for host prediction of φPDS1. FDR: False Discovery Rate.

**Table S4**. Intergenomic similarity values determined by VIRIDIC of φPDS1 (MN929097.1), 313 complete phage genomes from the candidate family *Paboviridae* and the isolated phages from Shen et al. (2023) study, PD491P1 (OP172815.1) and AS73P1 (OP172640.1).

**Table S5.** Genus clusters obtained by VIRIDIC after analyzing φPDS1 (MN929097.1) with 313 complete phage genomes from the candidate family *Paboviridae* and the isolated phages from Shen et al. (2023) study, PD491P1 (OP172815.1) and AS73P1 (OP172640.1).

**Table S6.** Genome length, percentage of GC content, and percentage of similarity compared to φPDS1 of the phages clustered within the new candidate genus *Sagittacolavirus* obtained by VIRIDIC analysis.

**Table S7.** Human gut metagenomic samples obtained from five different publicly available studies used to investigate the prevalence of φPDS1 in the human gut. A φPDS1 genome with a coverage of more than 30% was considered to be present in the metagenomic samples.

**Supplementary Figures**

Supplementary Figure 1.

**
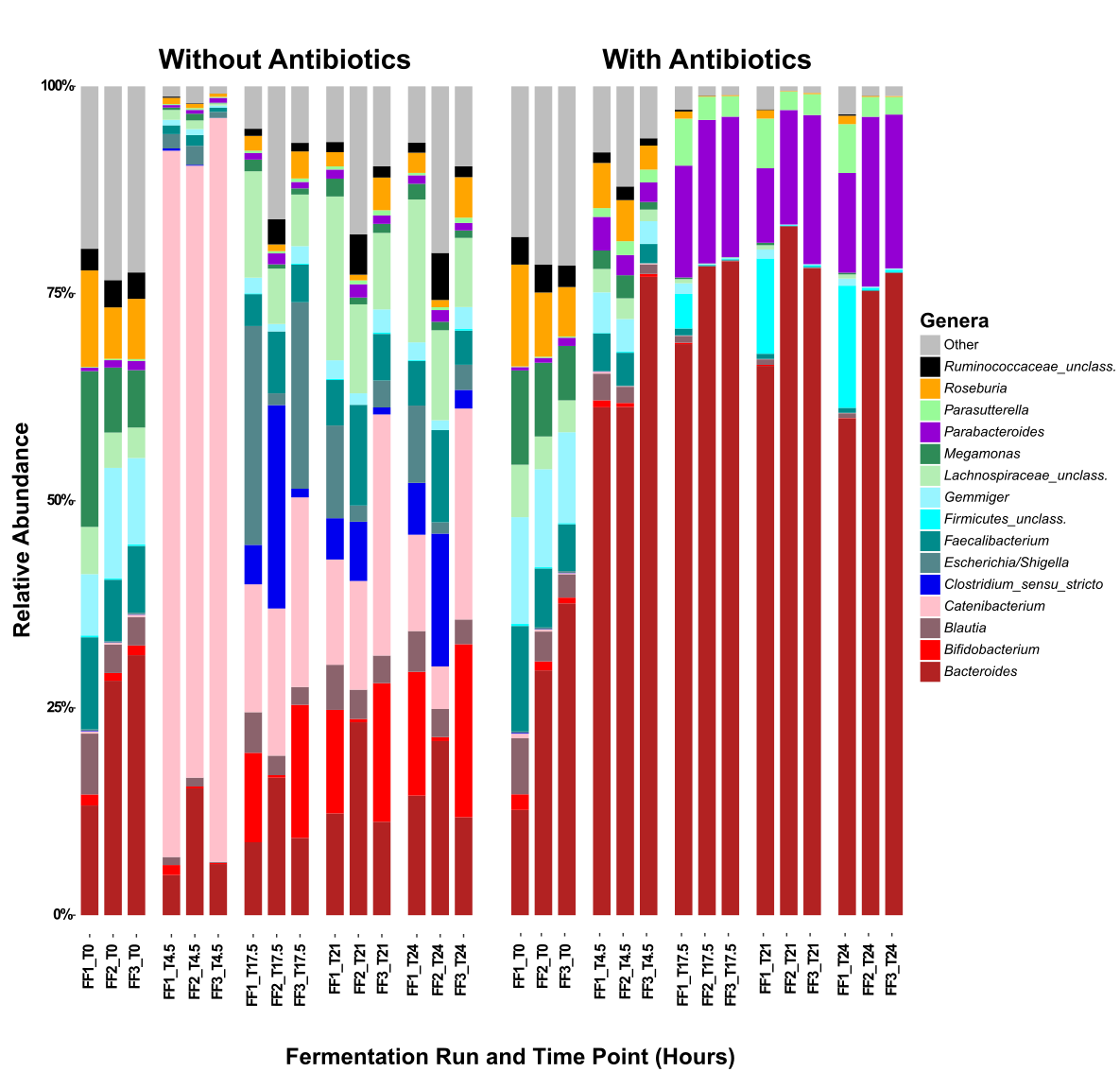
**

Supplementary Figure 2.

Supplementary Figure 3.

**
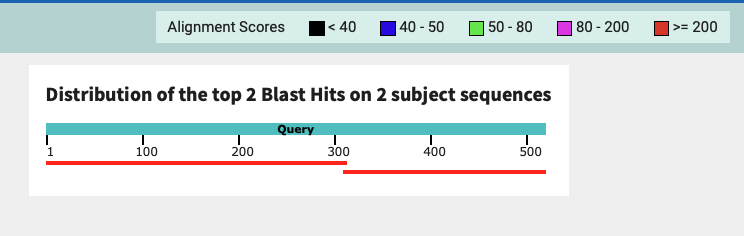
**

Supplementary Figure 4.

**
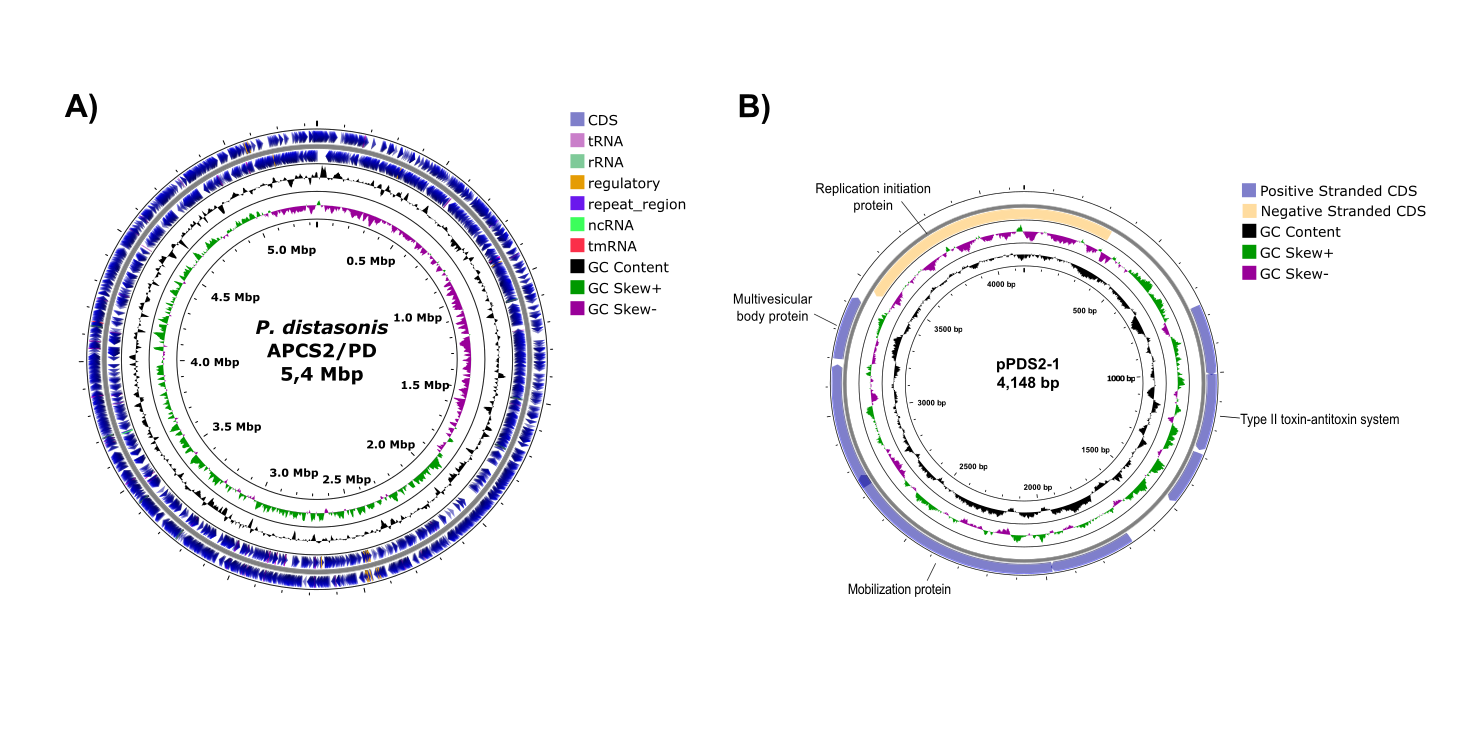
**

Supplementary Figure 5.


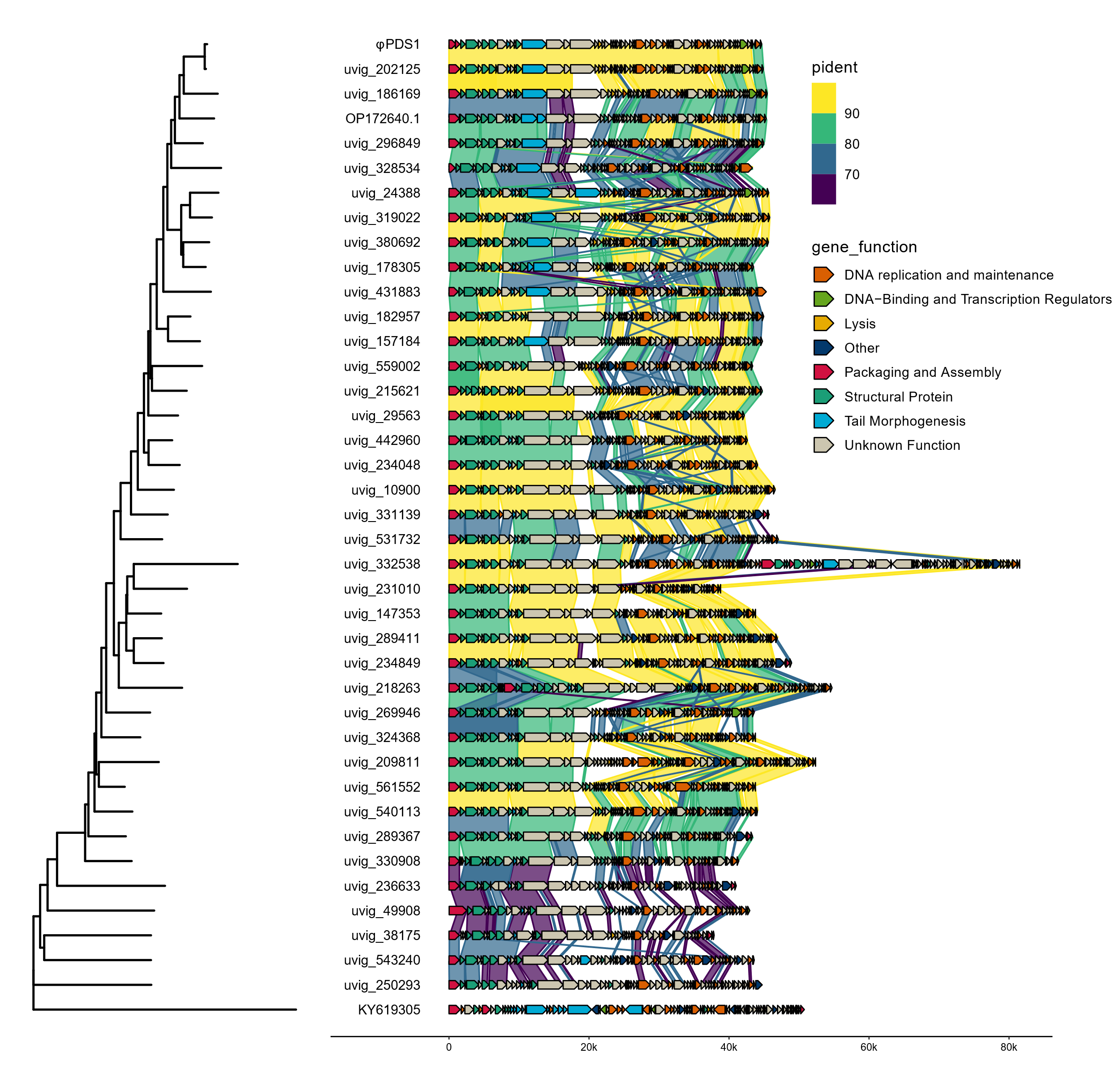


Supplementary Figure 6.


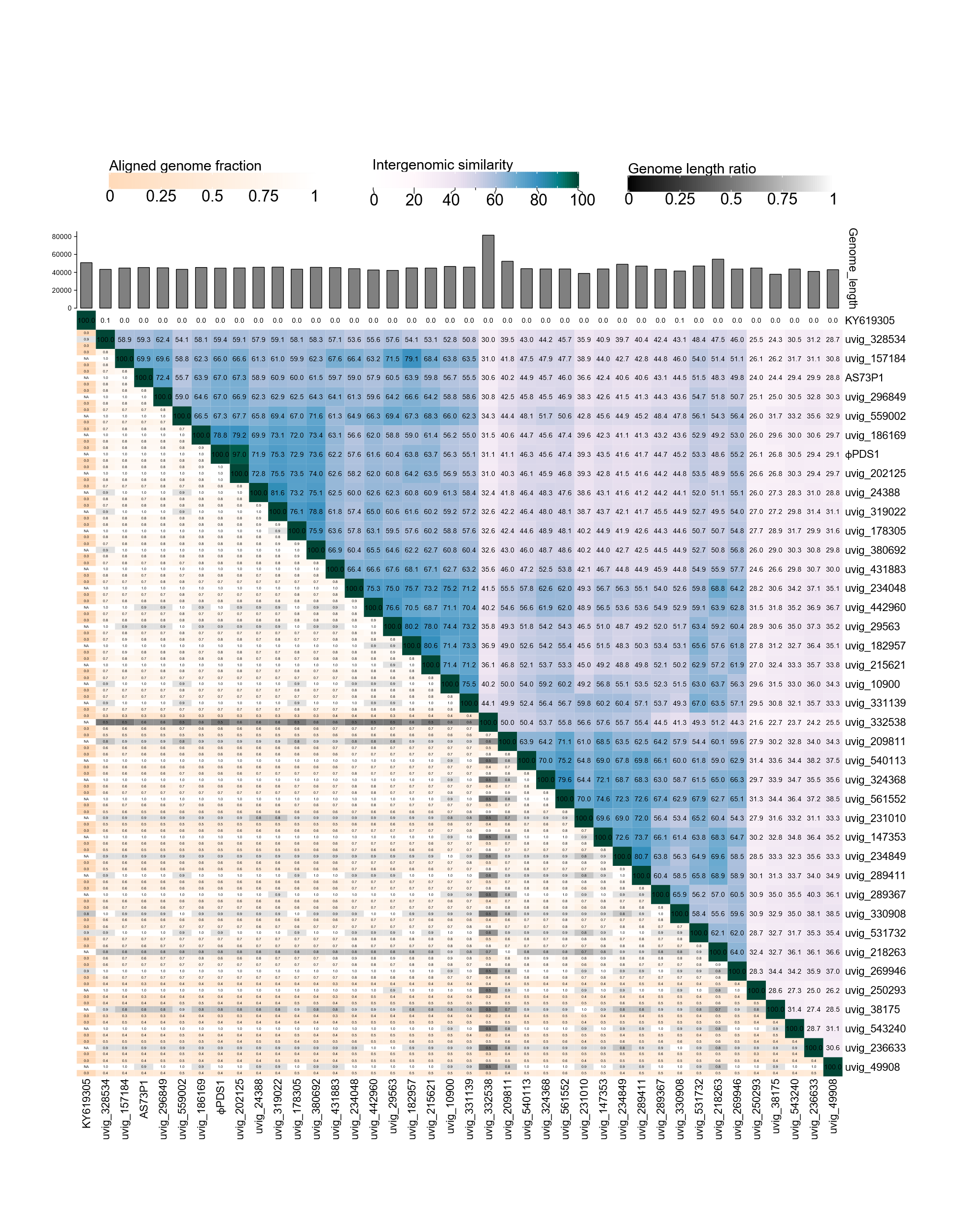


**Supplementary Figure 1.** Relative abundance of bacterial genera in vessels with and without selective antibiotics enrichment at each time point of the fermentation (0 h, 4.5 h, 17.5 h, 21 h and 24 h) to promote Bacteroidales expansion.

**Supplementary Figure 2.** Spot assay on the lawn of *P. distasonis* APCS2/PD. Samples spotted are as follows: **(A)** Faecal filtrate prepared from subject ID: 924 faeces. **(B)** Phage-rich fermenter filtrate collected at time point 21 hours. Selective enrichment to promote the growth of Bacteroidales was not performed in the case of A + B. **(C)** Phage-rich fermenter filtrate collected at time point 21 hours following selective enrichment.

**Supplementary Figure 3.** Blastp comparison between the large terminase (terL) protein identified in φPDS1 (red line) and the terL protein identified in uvig_202125 (query sequence). uvig_202125 is the phylogenetically closest identified phage sequence to φPDS1 in this study (Figure 4 and Table S5). The two red lines correspond to the terL proteins of φPDS1, indicating a complete alignment with uvig_202125 but with a split into two sequences. In contrast, uvig_202125 shows a single, uninterrupted sequence.

**Supplementary Figure 4. (A)** Circular map of the φPDS1 host genome, *P. distasonis* APCS2/PD, with a size of 5.35 Mbp, and **(B)** its associated plasmid pPDS2-1 with a size of 4,418 bp. The innermost circle (green and purple) depicts the GC skew, while the black circle represents the GC content. Blue arrows in the *P. distasonis* genome and blue and yellow arrows in the plasmid display the open reading frames identified on the positive (external) and negative (inner) DNA strands.

**Supplementary Figure 5.** Whole genome comparisons of φPDS1 against members of the different genus clusters within the candidate *Paboviridae* family identified by VIRIDIC, along with one outgroup member from the *Drexlerviridae* family (KY619305; Nouzillyvirus ESCO41). The figure shows the percentage of identity of different genomes compared to the closest phylogenetic phage genome at the nucleotide level. The comparison was performed using BLASTn. The colour between two genomes correspond to the percentage of identity between both genomes at the nucleotide level. The colour of the gene is based on the annotation given by Pharokka. The left-side tree was inferred using the VICTOR tool and the figure was created using the R packages gggenomes and ggtree.

**Supplementary Figure 6.** Heatmap generated by VIRIDIC illustrates the intergenomic similarity values (right half) and alignment indicators (left hand) between φPDS1, members of the different genus cluster (38) from the candidate family *Paboviridae* identified by VIRIDIC, and one *Drexlerviridae* phage used as an outgroup member (KY619305; Nouzillyvirus ESCO41).
